# IgG Conformer's Binding to Amyloidogenic Aggregates

**DOI:** 10.1371/journal.pone.0137344

**Published:** 2015-09-14

**Authors:** Monichan Phay, Alfred T. Welzel, Angela D. Williams, Helen P. McWilliams-Koeppen, Veronika Blinder, Tiernan T. O'Malley, Alan Solomon, Dominic M. Walsh, Brian O'Nuallain

**Affiliations:** 1 The Laboratory of Neurodegenerative Research, Brigham and Women’s Hospital, Harvard Institutes of Medicine, Boston, Massachusetts, United States of America; 2 The Conway Institute, University College Dublin, Belfield, Dublin, Republic of Ireland; 3 Human Immunology and Cancer Program, University of Tennessee Graduate School of Medicine, Knoxville, Tennessee, United States of America; Institut National de la Santé et de la Recherche Médicale (INSERM), FRANCE

## Abstract

Amyloid-reactive IgGs isolated from pooled blood of normal individuals (pAbs) have demonstrated clinical utility for amyloid diseases by *in vivo* targeting and clearing amyloidogenic proteins and peptides. We now report the following three novel findings on pAb conformer's binding to amyloidogenic aggregates: 1) pAb aggregates have greater activity than monomers (HMW species > dimers > monomers), 2) pAbs interactions with amyloidogenic aggregates at least partially involves unconventional (non-CDR) interactions of F(ab) regions, and 3) pAb's activity can be easily modulated by trace aggregates generated during sample processing. Specifically, we show that HMW aggregates and dimeric pAbs present in commercial preparations of pAbs, intravenous immunoglobulin (IVIg), had up to ~200- and ~7-fold stronger binding to aggregates of Aβ and transthyretin (TTR) than the monomeric antibody. Notably, HMW aggregates were primarily responsible for the enhanced anti-amyloid activities of Aβ- and Cibacron blue-isolated IVIg IgGs. Human pAb conformer's binding to amyloidogenic aggregates was retained in normal human sera, and mimicked by murine pAbs isolated from normal pooled plasmas. An unconventional (non-CDR) component to pAb's activity was indicated from control human mAbs, generated against non-amyloid targets, binding to aggregated Aβ and TTR. Similar to pAbs, HMW and dimeric mAb conformers bound stronger than their monomeric forms to amyloidogenic aggregates. However, mAbs had lower maximum binding signals, indicating that pAbs were required to saturate a diverse collection of binding sites. Taken together, our findings strongly support further investigations on the physiological function and clinical utility of the inherent anti-amyloid activities of monomeric but not aggregated IgGs.

## Introduction

Alzheimer's disease (AD) is the most common of ~30 amyloid disorders that are currently incurable and often fatal. These diseases involve the extracellular self aggregation of a peptide or protein that forms amyloid deposits on organ(s) [1, 2]. Amyloid deposits consist of β-sheet rich amyloid fibrils and accessory molecules [2, 3]. AD is a particularly complex disease since it involves the aberrant aggregation of amyloidogenic amyloid β peptides (Aβ) and the microtuble-associated tau protein [2, 4–6]. Other debilitating amyloid disorders, are caused by mutant and wild-type forms of a blood transport protein transthyretin (TTR) that primarily deposit in the heart and/or nerves [7–10].

Passive vaccination with humanized anti-amyloid monoclonal antibodies (mAbs) is a primary immunotherapeutic approach for amyloid diseases [11–13]. A recent novel therapeutic approach for AD has been to boost a patient's pool of amyloid-reactive IgGs using human intravenous immunoglobulin (IVIg). IVIg contains a diverse repertoire of pooled polyclonal human IgGs (pAbs), including anti-amyloid IgGs, from plasmas of 1000’s of normal individuals [14–16]. The rational for using IVIg for AD is their ability to reduce levels of soluble cerebral Aβ while increasing the peptide's blood pool [17, 18]–a process consistent with beneficial anti-Aβ immunotherapy [11, 17, 18]. *In vitro* and transgenic mice studies indicate that Aβ-reactive IVIg IgGs have therapeutic potential for AD [18–26]. Moreover, we have demonstrated that Aβ-reactive IVIg IgGs are cross-reactive against conformational epitopes on other amyloidogenic proteins and peptides. Thus, anti-amyloid pAbs isolated from normal human blood have demonstrated therapeutic potential not only for AD but for other amyloid diseases [20, 21, 27].

Recently, IVIg was tested in a 18-month phase 3 clinical trial for mild to moderate AD. The antibody did not meet its primary endpoints, but subgroup analysis indicated that IVIg had a slight beneficial effect for AD patients that were ApoE4 carriers and had moderate disease [28]. Presumably, IVIg's ineffectiveness may have been because its anti-amyloid activity was not potent enough, and patients may have benefited more from an IVIg-like preparation that had enhanced activity [29]. However, the development of a more viable and potent therapeutic reagent than IVIg has been hampered by our current poor understanding on its anti-amyloid activity. For example, it has been assumed, and not yet proven, that natural IgGs are the amyloid-reactive species in IVIg. To address this, we have now compared the anti-amyloid activities of IgG conformers (monomer, dimer, and HMW aggregates) contained in IVIg with conformers present in preparations of pAbs isolated from normal human and murine plasmas, and control mAbs generated against non-amyloid targets. Our findings strongly indicate that an IgG's anti-amyloid activity is enhanced when they aggregate (Dimers and HMW species), and is an intrinsic property that likely has physiological and clinical significance.

## Materials and Methods

### Proteins, peptides, and chemicals

Wild-type human Aβ1–40 (Aβ), DAEFRHDSGY-EVHHQK LVFF-AEDVGSN KGA-IIGLMVGGVV, and Aβ in which serine 26 was substituted with cysteine were synthesized and purified by Dr. James I Elliott at Yale University (New Haven, CT). Peptide masses and purities (>95%) were confirmed by electrospray ionization/ion trap mass spectrometry and reverse-phase HPLC. Aβ concentration was determined by absorbance at 275 nm using the molar extinction coefficient for tyrosine (ε_275_ = 1400 M^-1^ cm^-1^).

Recombinant wild-type human TTR that was >95% pure by SDS-PAGE was obtained from Athens Research & Technology Inc. (Athens, GA). TTR's concentration was determined by absorbance at 280 nm using an E_280_^0.1%^of 1.35 [30]. A pure (>95% by SDS-PAGE) recombinant form of a methionylated N-terminal 165 amino acid fragment of human vascular endothelial growth factor A (VEGF-165) was purchased from BioLegend Inc. (San Diego, CA). A hydrophobic maize protein, zein, bovine elastin, human DNA, murine extracellular matrix gel, and chicken ovalbumin were from Sigma-Aldrich (Saint Louis, MO, USA).

Preparations of IVIg (Gammagard liquid®) were provided by Baxter BioScience (Vienna, Austria). Sterile-filtered pooled normal human and murine plasmas were obtained from Equitech-Bio Inc. (Kerriville, TX). Human myeloma mAbs were from Binding Site Group Ltd (Birmingham, UK). Human IgG_1_ mAbs, Avastin (anti-VEGF [31]) and Synagis (anti-human respiratory syncytial virus [32]), were from Genentech, Inc. (San Francisco, CA) and MedImmune LLC (Gaithersburg, MD), respectively. Murine anti-Aβ mAb, 6E10 [33], and anti-TTR mAb, 9G6 [34], were from Signet (Dedham, MA)) and Thermo Fisher Scientific Inc. (Waltham, MA), respectively. An anti-HIV-1 gp120 IgG_1_ MAb, 46–4 [35], was produced in-house by high density growth of the hybridoma (American Type Culture Collection, Manassas, VA) in a 1000 mL CELLine bioreactor flask (CL1000, Integra Biosciences AG, Chur, Switzerland). MAb 46–4 was purified from hybridoma supernatants using a 1 mL HiTrap protein A HP column (GE Healthcare, Uppsala, Sweden). MAb 46–4's concentration was determined by absorbance at 280 nm using an extinction coefficient (E_280_^0.1%^) of 1.4 [36]. Size exclusion chromatography (SEC) protein standards were obtained from Bio-Rad (Hercules, CA). All other chemicals were obtained from Sigma-Aldrich and were of the highest purity available.

### Preparation of amyloidogenic conformers

WT Aβ monomers and fibrils, disulfide cross-linked S26C Aβ dimers and protofibrils generated from S26C Aβ dimers (PFs), WT TTR fibrils and soluble TTR aggregates (SAgg) were generated and characterized as previously described [34, 37]. Briefly, pooled SEC fractions of Aβ monomers (~0.1 mg/mL in 25 mM ammonium acetate, pH 8.5) was generated from ~1 mg/mL Aβ in 50 mM Tris containing 6M guanidine HCl, pH 8.2 that was loaded onto a Superdex™ 75 10/300 GL column (GE Healthcare Bio-Sciences AB, Uppsala Sweden) equilibrated in 25 mM ammonium acetate, pH 8.5. Purity of Aβ monomer fractions was determined by SDS-PAGE [37]. WT Aβ fibrils were generated from ~0.2 mg/ml of Aβ monomers in PBS containing 0.02% sodium azide, pH 7.4, by incubating the peptide at 37°C for 14 days. Fibrillogenesis was judged complete when Thioflavin T (ThT) fluorescence had reached a maximum plateau value. The reaction product was harvested by centrifugation at 20,200 x g for 30 min at room temperature, and fibril morphology confirmed by negative contrast EM [37]. Pooled SEC fractions of S26C Aβ dimers (~0.1 mg/mL in 25 mM ammonium acetate, pH 8.5) were generated from ~1 mg/mL of oxidized S26C Aβ in 50 mM Tris-HCl containing 6 M guanidine HCl, pH 8.0, that was loaded onto a HiLoad 16/60 Superdex 75 column (GE Healthcare Bio-Sciences AB) equilibrated with 25 mM ammonium acetate, pH 8.5. Purity of S26C Aβ dimer fractions was determined by SDS-PAGE [37]. PFs were generated from S26C Aβ dimers by a 1:1 dilution of the dimeric peptide in 25 mM ammonium acetate, pH 8.5, into 2X PBS, pH 7.4, and incubating the peptide at 37°C for ~3 days until a maximum ThT fluorescence plateu value was obtained [37]. PFs morphology in the reaction product was confirmed by EM and by the retention of ThT-positive Aβ in sample supernatants after bench-top centrifugation (16,000 x g for 20 min) [37]. WT TTR fibrils were generated by incubating ~0.5 mg/mL of the native protein for 4 days at 37°C in 400 mM sodium acetate, pH 4.0. Fibrillogenesis was judged complete by ThT fluorescence, and the fibrillar product harvested and fibrillar morphology confirmed by EM [34]. SAggs were generated by pelleting WT TTR fibrils, removing the supernatant, and re-solubilizing the fibrillar protein by adding 0.1% ammonium hydroxide to the same volume as the discarded supernatant [34]. After ~5min, the solution was neutralized with the same volume of 0.5 M Tris-HCl (pH 7.0) and centrifuged to remove any trace insoluble aggregates. Amyloid-like soluble aggregates in the final preparation was confirmed by ThT fluorescence and size-exclusion chromatography using a Superdex™ 75 10/300 GL column (GE Healthcare Bio-Sciences AB).

Immediately before use, soluble Aβ and TTR conformers were centrifuged, 16,000 x g for 20 min at 4°C, to remove any contaminating insoluble aggregates. Native tetrameric TTR was SEC-purified from trace aggregates using a Superdex 75™ 10/300 GL column (GE Healthcare, Uppsala, Sweden) equilibrated with PBS, pH 7.4. Amyloidogenic conformers were stored in working aliquots at -80°C for up to ~3 months.

### Electron microscopy

Negative contrast electron microscopy was performed as described previously [37]. Briefly, 10 μl aliquots of each test sample were applied onto duplicate carbon-coated formvar grids (Electron Microscope Sciences, Washington, PA), cross-linked with 0.5% (v/v) glutaraldehyde (Ted Pella Inc., Redding, CA), and stained with 2% (w/v) uranyl acetate solution (Ted Pella Inc.). The grids were examined using a Tecnai™ G^2^ Spirit BioTWIN electron microscope (FEI, Hillsboro, OR).

### IgG Purification from normal mammalian plasma

Three different purification methods (protein A, protein G and Melon™ gel chromatography) were used to identify an optimal procedure for isolating pAbs from pooled normal human and murine plasmas. Protein G purification of pAbs involved diluting plasma 1:5 into binding buffer (PBS, pH 7.4), passing the sample over a HiTrap protein G HP column (GE Healthcare), washing the column with binding buffer, and eluting column-bound IgGs with 100 mM Glycine-HCl, pH 2.7, into eppendorf tubes that contained neutralization buffer (1 M Tris, pH 9.0). Protein A isolation of human pAbs involved a 1:5 into dilution of plasma into binding buffer (PBS, pH 7.4), passage over a HiTrap protein A HP column (GE Healthcare), and elution with 0.1 M glycine, pH 3.5, into eppendorf tubes containing neutralization buffer. Except for binding buffer consisting of 1.5 M glycine containing 3 M NaCl, pH 8.9, protein A-isolation of murine pAbs was carried out in the same manner as for human pAbs. Melon™ gel isolation of mammalian pAbs from pooled plasmas was determined using 1 mL Melon™ gel columns (Thermo Fisher Scientific Inc.) as per manufacturer’s instructions. To minimize IgG aggregation, all purified antibodies were immediately buffer exchanged to a final concentration of ~1 mg/mL in PBS, pH 7.4, using 40 kDa Zeba spin columns (Thermo Fisher Scientific Inc.). Antibodies were used immediately, or stored in working aliquots at -20°C. IgG concentrations were determined by absorbance at 280 nm using an extinction coefficient (E_280_^0.1%^) of 1.4. IgG purities were established by SDS-PAGE and SEC using a HiPrep 16/60 Sephacryl S-300 HR column (GE Healthcare).

### Dynamic light scattering (DLS)

DLS was used to estimate the size of IgG conformers present in unfractionated, SEC- and affinity column-fractionated IVIg, pAb and mAb samples. DLS was carried out at room temperature using a DynaPro plate reader (Wyatt Technologies Corp., Santa Barbara, CA) [38]. Briefly, 100 μl aliquots of ~0.02 to ~1 mg/ml antibody samples and buffer blanks were added to wells of a polystyrene flat bottom 96 well half-area black microplate (cat# 3881, Corning, Tewksbury MA). DLS readings were performed using 6 to 20 10s acquisitions with auto-attenuation. The hydrodynamic radius (*R*_*h*_), polydisperse indice, and apparent molecular weights for IgG conformers in each sample were calculated using DYNAMICS V6 software (version 7.1.7.16, Wyatt, Santa Barbara, California).

### Affinity purification of anti-amyloidogenic IgGs

Anti-amyloid IgG conformers were isolated from preparations of IVIg and mAbs using Aβ and Cibacron blue affinity chromatography, as described [20, 27]. The Aβ column consisted of S26C Aβ that was solubilized with 0.1 NH_4_OH and then aggregated at ~0.2 mg/mL in PBS, pH 7.4, for 5 days at 37°C. DLS analysis was used to estimate IgG conformer sizes in column eluants. Affinity purified IgGs were used instantly for ELISA and/or SEC studies.

### Protein aggregation

IgG aggregates were generated by heating ~4 mg/mL of SEC-isolated monomeric IVIg or mAb Avastin in PBS, pH 7.4, in a water bath at 71°C for up to 30 min [39, 40]. Reactions were stopped by transferring heated samples to ice when light scattering at A_400nm_ had reached ~0.6 or ~3.9 [39]. Soluble IgG aggregates were separated from insoluble species by centrifuging reaction samples at ~16,000 x g for 25 min at room temperature, and collecting the resultant supernatants and pellets. IgG pellets were resuspended at ~2 mg/mL in PBS, pH 7.4. The amount of IgG conformers in each sample was determined by SDS-PAGE, MicroBCA assay (ThermoFisher Scientific Inc), and SEC. Amorphous aggregates of chicken ovalbumin were generated by iodoacetic acid carboxymethylation of the reduced and denatured protein, as described [41].

### SEC Purification of IgG conformers

Antibody monomers, dimers, and high molecular weight (HMW) aggregates were isolated from preparations of IVIg, pAbs and mAbs using a HiPrep 16/60 Sephacryl S-300 HR or a Superdex 200 Increase 10/300 GL column (GE Healthcare). The columns were equilibrated in PBS, pH 7.4, and calibrated using gel filtration protein standards (Bio-Rad). The size of IgG conformers present in SEC fractions was estimated by DLS. SEC fractionated IgGs were used immediately for ELISA or for generating heat-induced antibody aggregates.

### Antibody fragments

F(ab), F(abˈ)_2_, and Fc fragments of IVIg were generated using antibody fragmentation preparation kits, as per manufacturer's instructions (cat#s 44985 and 44988; Pierce Biotechnology, Rockford, IL). Briefly, papain generated F(ab) and Fc fragments were produced by incubating ~10 mg/ml IVIg in Fab digestion buffer (Pierce Biotechnology), pH 10, with papain-immobilized agarose for 5 h at 37°C. F(ab) fragment reaction product was separated from Fcs and undigested IgGs using 1 mL NAb™ Protein A Plus Spin columns (Pierce Biotechnology). Pepsin generated F(abˈ)_2_ fragments were produced by incubating ~10 mg/ml IVIg in F(abˈ)_2_ digestion buffer (Pierce Biotechnology), pH 4.4, with pepsin-immobilized agarose for 24 h at 37°C. F(abˈ)_2_ fragments were purified from undigested IgG and digested Fc using 1 mL NAb™ Protein A Plus Spin columns. IgG fragments were confirmed to be >95% pure by non-reducing SDS-PAGE and by ELISA, using anti-Fc (Jackson ImmunoResearch Laboratories Inc., West Grove, PA) and anti-γ chain (Sigma-Aldrich) secondary antibodies. IgG fragments were used immediately or stored in working aliquots at 4°C for up to 1 month.

### Antibody binding to amyloidogenic conformers-ELISAs

Direct and competition ELISAs were the primary methods used to establish IgG binding to Aβ and TTR conformers [21, 27, 41]. ELISA experiments were carried out in duplicate with 1% BSA in PBS, pH 7.4, as blocking buffer, and assay buffer consisting of blocking buffer containing 0.05% tween 20. The detection system consisted of a biotinylated goat anti-human or anti-mouse IgG (γ-specific, Sigma-Aldrich), streptavidin-horse radish peroxidase (Jackson ImmunoResearch Laboratories, Inc.), and colorimetric TMB substrate (SureBlue ReserveTM; KPL, Gaithersburg, MD, USA). Alternatively, plate-bound IgG was detected using the biotinylated secondary antibodies with strepetavidin-Eu^+3^ and enhancement solution (DELFIA^®^; PerkinElmer, Inc., Boston, MA, USA) [21, 41]. Absorbance (A_450nm_) and Eu^+3^ time-resolved fluorescence (Ex_340nm_/Em_615nm_) assay signals were measured using a SpectraMax M2 multi-detection (Molecular Devices Corp., Sunnyvale, CA) and a Wallac Victor 2 (PerkinElmer, Inc.) microtiter plate reader, respectively. Antibody binding and competition curves were generated by subtracting background controls from assay signals. The resultant graphs were fit using a standard 3-parameter sigmoid (logistic) function (SigmaPlot 2000, version 6; Systat Software, Chicago, IL). The concentration of antibody that gave half-maximal binding, EC_50_, and the concentration of competitor that gave half maximal inhibition, IC_50_, were determined from the fitted curves.

The ability of human and murine pAbs to compete with each other for the same or similar binding sites on amyloidogenic conformers was determined using a hybrid capture/competition ELISA. The assay established the inhibition of plate-immobilized IVIg F(ab) fragments (200 ng/well) binding to 8 μg/mL of solution-phase PFs by serially diluted (6 nM to 6 μM) human and murine pAb competitors. The detection system consisted of an in house pan-Aβ reactive polyclonal rabbit antibody, AW7 [42], a horse radish peroxidase-conjugated goat anti-rabbit IgG (GE healthcare), and TMB substrate (SureBlue ReserveTM; KPL). IC_50_ values were determined from the sigmoid-fitted curves.

### Immunoprecipitation/Western blots

IVIg, pAbs, and mAb binding to Aβ conformers was investigated using duplicate 0.5 ml reaction samples, as previously described [43]. Each sample consisted of 0.5 or 5 μg/mL of an Aβ conformer and 20, 100, or 200 μg/mL antibody. Positive control immunoprecipitation (IP) experiments were carried out using an in-house polyclonal pan-Aβ reactive antibody, AW8 [27, 42]. Except for the absence of protein G beads or test antibody, negative control IPs were setup in the same manner as for test IPs. All samples were incubated for 1 h at room temperature, beads centrifuged at 4,000 x g for 5 min, pellets washed with IP STEN buffers, and antibody-bound Aβ liberated by heating the pellets at 100°C for 5 min in 2 x SDS sample buffer [43]. The boiled samples were electrophoresed on 16% polyacrylamide tris-tricine gels, transferred onto 0.2 μm nitrocellulose (Optitran, Schleicher and Schull, Germany) at 400 mA for 2 h. Membrane-bound Aβ was detected by enhanced chemiluminescence (Thermo Fisher Scientific Inc.) using 1 μg/mL of an N-terminal Aβ-reactive mAb 6E10 (7 nM) [43]. In addition, mAb Avastin's ability to IP Aβ in the presence of a 5 molar excess (with respect to Avastin) of a N-terminal 165 amino acid fragment of its immunogen VEGF (VEGF-165; BioLegend Inc.) was determined by Western blots against Aβ, as above, and VEGF-165-bound Avastin, using 1 μg/mL Avastin (7 nM) as the primary antibody.

## Results

### Human and murine pAbs similarly cross-react with amyloidogenic aggregates

**Fig 1:**
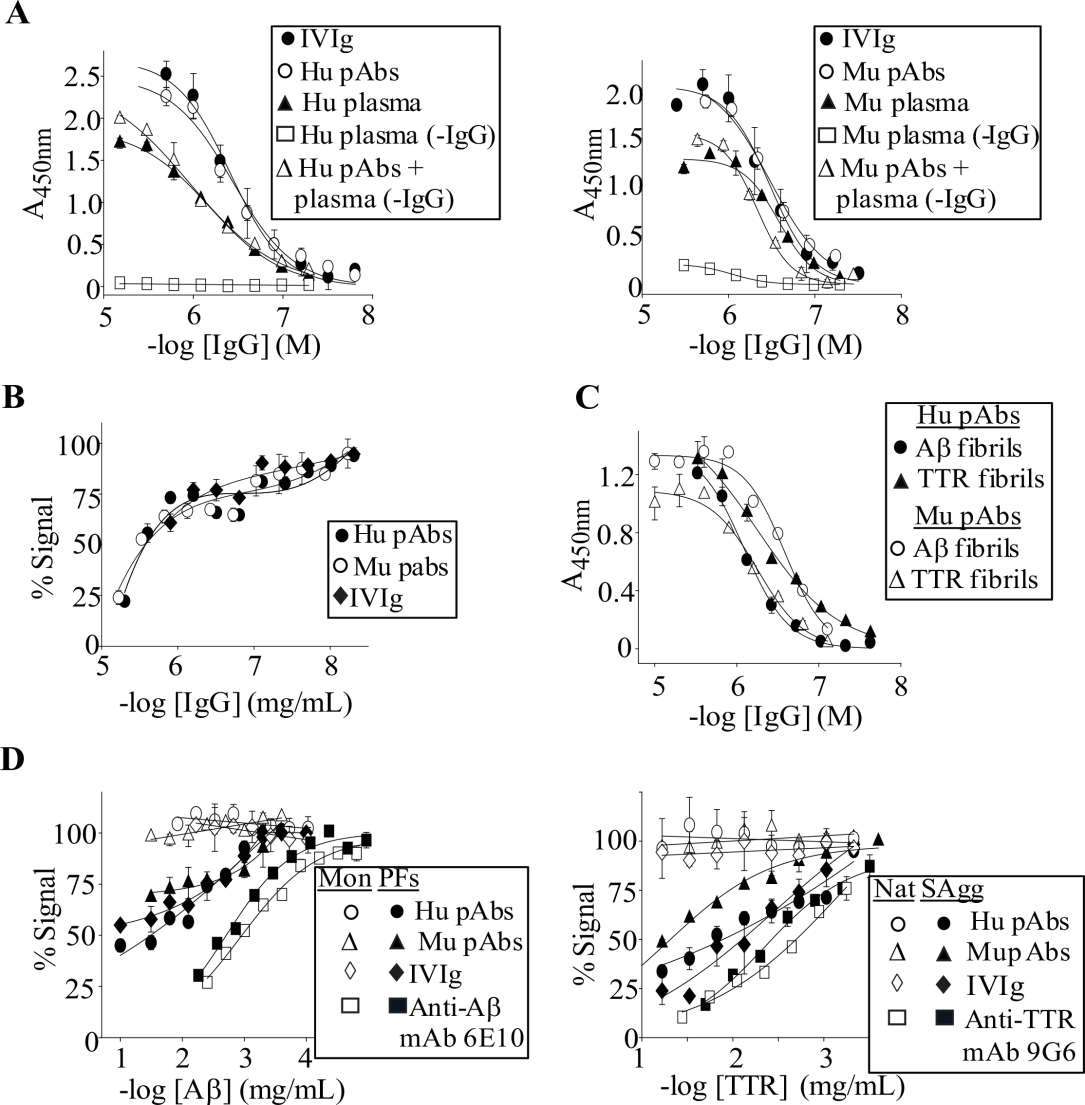
IVIg and Protein A-purified human and murine pAbs have similar anti-amyloid activities. (**A**) IgG binding curves against plate-immobilized PFs for IVIg and protein A-purified human (Hu) and murine (Mu) pAbs from pooled normal plasmas. Antibody binding curves are also shown for pAbs present in or dosed back into plasma. (**B**) Hybrid capture/competition ELISA curves for pAb's and IVIg's dose-dependent inhibition of PFs binding by plate-immobilized IVIg F(ab') fragments. The assay was carried out using 8 μg/ml solution-phase PFs. (**C**) Antibody binding curves for Hu and Mu pAb's nM cross-reactivity with plate-immobilized Aβ and TTR fibrils. (**D**) Left panel: Competition curves for solution-phase PF's and Aβ monomer's (Mon) inhibition of pAbs and IVIg binding to plate-immobilized PFs. Right panel: SAgg's and native TTR's (Nat) inhibition of pAbs and IVIg binding to plate-immobilized TTR fibrils. Competition studies were carried out using IgG concentrations, ~500 nM, which were equivalent to their EC_50_ values for binding to PFs or TTR fibrils. Each binding or competition curve was carried out in duplicate, and bars represent the standard errors.

Fig 1 and Table 1 show that protein A-purified pAbs from pooled normal human plasmas and IVIg had essentially the same reactivity with plate-immobilized PFs, with EC_50_s of ~300 nM. Given pAb preparations each consisted of a heterogenous mix of IgGs, each EC_50_ value that was determined was the “apparent” activity for all IgG types in the preparation. The purified IgGs had the same activity as pAbs present in, or dosed back into pooled normal plasmas (Fig 1A). The later findings confirmed that protein A was an optimal method for purifying pAbs since it did not modulate their avidities for PFs. SDS-PAGE and SEC confirmed that our pAb preparations were highly pure (~95%), and essentially free of plasma IgMs. Nevertheless, given IgMs can bind to protein A [44], we ensured that any contaminating IgMs did not influence our ELISA measurements of pAb’s binding to PFs and other amyloidogenic conformers by using secondary antibodies that were specific for human and murine IgGs (γ heavy chain specific). In contrast to protein A-isolated pAbs, protein-G purified pAbs had ~5-fold enhanced PFs reactivity, and Melon gel-isolated pAbs were not sufficiently pure (<70% pure) to carry out PFs binding studies. Presumably, the protein G-isolated pAb's enhanced activity was due to the low pH elution buffer's (0.1 M glycine HCl, pH 2.7) artifactual enhancement of the IgG's polyreactivity [45].

**Table 1:** Mammalian IgGs binding to plate-immobilized amyloidogenic conformers. Each value for EC_50_ and maximum assay signal amplitude was determined from the average of two to four sigmoidal fitted antibody binding curves, as shown in Figs 1, 2 and 7.

		PFs		SAgg	
IgG	Preparation	EC_50_ (nM)	Max. Signal (A_450nm_)	EC_50_ (nM)	Max. Signal (A_450nm_)
Human pAbs	Unfractionated	363 ± 3.3	2.5 ± 0.1	619 ± 24	1.2 ± 0.1
	SEC mon^1^	1206 ± 45	2.8 ± 0.3	n.d.^3^	n.d.
Mouse pAbs	Unfractionated	331 ± 4.6	2.1 ± 0.2	261 ± 3.4	1.3 ± 0.2
	SEC mon	698 ± 18.6	3.2 ± 0.4	n.d.	n.d.
IVIg	Unfractionated	269 ± 2.7	2.4 ± 0.2	250 ± 2.0	1.4 ± 0.1
	SEC mon	576 ± 26	2.6 ± 0.1	481 ± 4.0	n.d.
	SEC dimer^2^	81 ± 1.0	3.2 ± 0.4	131 ± 1.2	n.d.
Synagis	Unfractionated	891 ± 13	0.8 ± 0.3	2523 ± 25	0.8 ± 0.1
mAb	SEC mon	1611 ± 30	0.3 ± 0.0	n.d.	n.d.
	SEC dimer	229 ± 4.6	~2.0	n.d.	n.d.
Avastin	Unfractionated	1870 ± 30	0.3 ± 0.0	4130 ± 87	0.7 ± 0.1
mAb	SEC mon	3273 ± 78	0.3 ± 0.0	n.d.	n.d.
	SEC dimer	185 ± 2.1	>0.7	n.d.	n.d.

^1,2^SEC-isolated IgG monomers (mon) and dimers as shown in Fig 2A.

^3^n.d. stands for not determined.

Given IVIg's anti-amyloid activity has been attributed to naturally occurring autoantibodies [23], we investigated if human pAb's anti-amyloidogenicity could be mimicked by protein A-isolated pAbs from normal murine plasmas. Fig 1A and Table 1 show that murine and human pAbs, and IVIg, had essentially the same binding to plate-immobilized PFs. Given the IgG's similar avidities, we used a hybrid capture/competition ELISA to establish if they competed for the same or similar binding sites. Fig 1B shows that all three IgGs similarly dose-dependently inhibited plate-immobilized IVIg F(ab)s binding to PFs, with IC_50_s of ~3 μM. The IgG's also cross-reacted with Aβ fibrils, TTR fibrils, and soluble TTR aggregates (SAgg), with EC_50_s in the 300–600 nM range (Fig 1C, Table 1). Given that surface-adsorbed amyloidogenic conformers can alter antibody reactivity [20, 46], we investigated if the IgG's could still bind to soluble amyloidogenic conformers (PFs and SAgg) in a solution-phase competition ELISA. Fig 1D shows that PFs, but not Aβ monomers, dose-dependently inhibited the pAbs and IVIg from binding to plate-immobilized PFs, with IC_50_s of ~10 μg/mL. In contrast, both Aβ monomers and PFs potently inhibited a pan-Aβ reactive murine mAb, 6E10 (Signet Laboratories), binding to plate-immobilized PFs (Fig 1D). Competition ELISA also demonstrated that the pAb's and IVIg's binding to plate-immobilized TTR fibrils were dose-dependently inhibited by solution-phase SAgg, but not by native TTR (Fig 1D). A ~4-fold lower inhibition was obtained for murine compared with human pAbs, with an IC_50_ of ~50 μg/mL. In contrast, a pan-TTR reactive mAb 9G6's (Thermo Fisher Scientific Inc.) binding to plate-immobilized TTR fibrils was inhibited by both native TTR and SAgg (Fig 1D).

### PAb binding to amyloidogenic aggregates is enhanced when pAbs aggregate, and involves F(ab)s of intact IgGs

**Fig 2:**
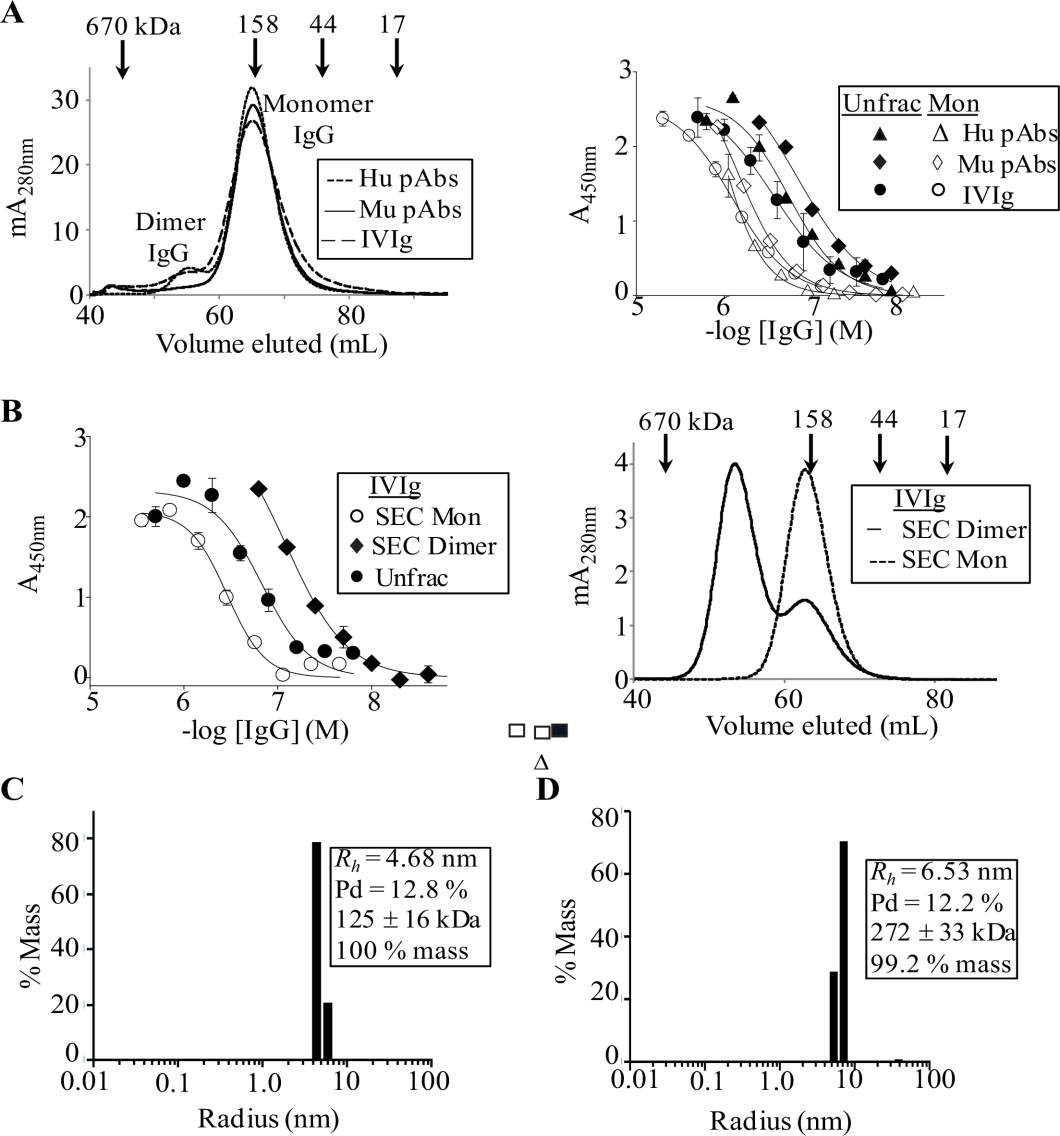
Dimeric pAbs bind more tightly to PFs than their monomeric form. (**A**) Left panel: SEC chromatograms for ~1 mg/mL of protein A-purified pAbs and IVIg in PBS, pH 7.4. SEC was carried out using a Hiprep16/60 Sephacryl S300 HR column (GE Healthcare) that was equilibrated in PBS, pH 7.4. Arrows indicate the elution of protein molecular weight standards. Right panel: Antibody binding curves against PFs for unfractionated (Unfrac) and SEC-isolated monomeric pAbs. (**B**) Left panel: Antibody binding curves against PFs for unfractionated and SEC-isolated monomeric and dimeric IVIg IgGs. Right panel: SEC chromatographs for ~0.2 mg/ml IVIg dimers and monomers in PBS, pH 7.4, after incubation under ELISA-like conditions (4 h at 37°C). (**C**) Dynamic light scattering for 0.8 mg/mL and 0.06 mg/mL of SEC-isolated IVIg monomers and (**D**) dimers, respectively. Dynamic light scattering was determined at room temperature immediately after SEC-isolation of each IgG conformer in PBS, pH 7.4. *R*_*h*_ and pd are abbreviations for hydrodynamic radius and polydispersity, respectively.

Having established that human and murine pAbs had similar avidities for amyloidogenic aggregates, we investigated if their activities depended on IgG conformer type (monomer, dimer, or high molecular weight (HMW) species). To do this, we SEC-isolated IgG conformers from preparations of pAbs and IVIg and established their binding to PFs. Fig 2A and Table 1 show that SEC-isolated pAbs and IVIg monomers were ~2- to 3-fold weaker than the unfractionated antibodies at binding to plate-immobilized PFs, respectively, with EC_50_s of ~600 nM to 1 μM (Table 1). SEC fractionation of pAbs and IVIg indicated that their relatively strong reactivity's with PFs was due to IgG dimers and/or trace amounts of HMW aggregates (Fig 2A). Moreover, SEC-isolated IVIg dimers bound 5- to 10-fold stronger than monomers to PFs and SAgg, with EC_50_s of ~80 and 130 nM, respectively (Table 1, Fig 2B). Dynamic light scattering (DLS) confirmed that IgG monomer and dimer samples were homogenous and >99% pure, with hydrodynamic radii (*R*_*h*_) of 4.68 nm (12.8% polydispersity) and 6.53 nm (12.2% polydispersity), respectively (Fig 2C and 2D).

**Fig 3:**
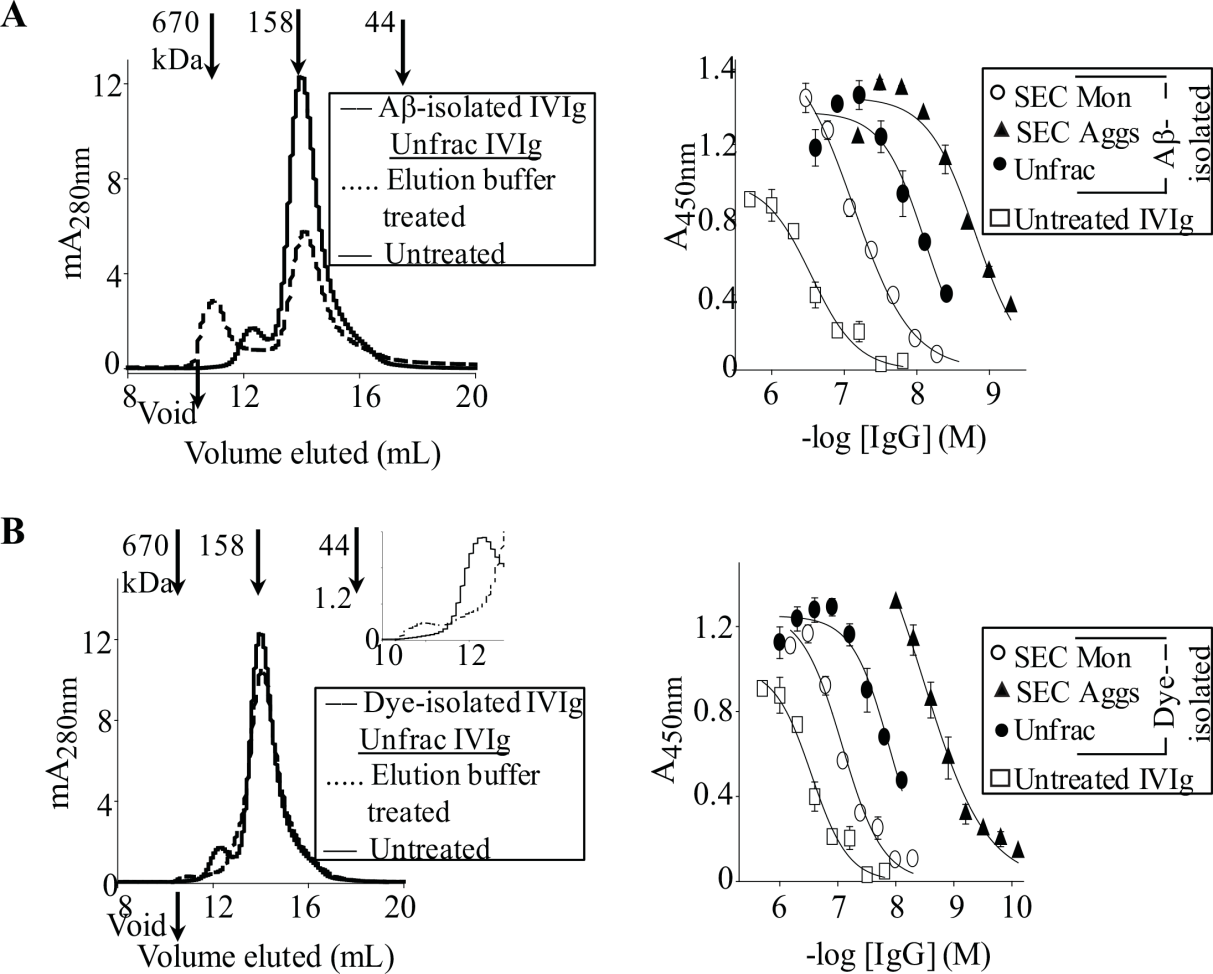
IgG aggregates are primarily responsible for the enhanced anti-amyloid activities of Aβ- and Cibacron blue-isolated pAb IgGs. (**A**) Left panel: SEC chromatograms for ~0.5 mg/mL of Aβ-isolated IVIg IgGs, and for IVIg, untreated, or diluted into column elution buffer (0.1 M glycine, pH 2.7) that was used to elute Aβ-bound IVIg IgGs. SEC was carried out using a Superdex 200 increase 10/300 GL column (GE Healthcare) that was equilibrated with PBS, pH 7.4. Right panel: IgG binding curves against plate-immobilized PFs for untreated IVIg, and for Aβ column-isolated IVIg IgGs that were used unfractionated (Unfrac) or as SEC-isolated monomers (SEC Mon) or aggregates (SEC Aggs). SEC Aggs consisted of a pool of IgG conformers (dimers and HMW species) that eluted before the monomeric antibody. (**B**) Left panel: SEC chromatograms for ~0.5 mg/mL dye-isolated IVIg IgGs, and for unfractionated IVIg that was untreated or diluted into column elution buffer (PBS containing 1.5 M NaCl, pH 7.4) that was used to elute dye-bound IVIg IgGs. Right panel: IgG binding curves against plate-immobilized PFs for unfractionated and SEC-isolated conformers of dye-isolated IVIg IgGs, and for untreated IVIg.

The enhanced binding of amyloidogenic aggregates by IgG aggregates was further evident from an enrichment of HMW IgG species in preparations of Aβ column-isolated IVIg IgGs. SEC chromatographs in Fig 3A show that Aβ-isolated IVIg IgGs, but not the unfractionated antibody, contained IgG aggregates that primarily eluted near the void volume of the Superdex 200 Increase 10/300 GL column (GE Healthcare). DLS on SEC fractions eluted near the column void volume (~10–12 mL) confirmed the presence of HMW IgG assemblies with a *R*_*h*_ of 59.1 nm (14.4% polydispersity). DLS analysis of pooled SEC fractions (~12–13 mL) collected nearer the IgG dimer peak, revealed the presence of two major species: 1) IgG dimers with an *R*_*h*_ of 6.7 nm that accounted for ~80% of the signal, and 2) HMW species with a *R*_*h*_ of 39 nm that accounted for 20% of the signal. Control experiments confirmed that IgG aggregates contained in Aβ-isolated IVIg IgG preparations were not induced by the low pH buffer (0.1 M glycine, pH 2.7) that was used to elute the antibodies off the Aβ column (Fig 3A).

ELISA studies with unfractionated and SEC fractionated IgG conformers of Aβ column-isolated IVIg IgGs confirmed that IgG aggregates were primarily responsible for the preparation's enhanced anti-amyloid activity. Fig 3A and Table 1 show that the ~40-fold enhanced PFs binding by Aβ-isolated IVIg IgGs compared with the unfractionated antibody was primarily due to a mixture of aggregate species (dimers and HMW species). The SEC-isolated IgG aggregates had ~180-fold stronger binding to PFs than unfractionated IVIg, with an EC_50_ of 1.6 ± 0.1 nM (Fig 3A, Table 2). Moreover, Aβ-isolated IVIg IgG monomers were ~11-fold stronger than monomers of unfractionated IVIg at binding to PFs, with an EC_50_ of 74 ± 1.0 nM (Fig 3A, Tables 1 and 2). DLS confirmed that Aβ-isolated IgG monomers were highly pure (99.5%), but contained 0.5% of HMW aggregates with a *R*_*h*_ of 12.1 nm (8.9% polydispersity).

**Table 2:** Unfractionated, Aβ- and Cibacron blue-isolated human IgGs binding to plate-immobilized PFs. Each value for EC_50_ and maximum signal amplitude was determined from the average of two to three sigmoidal fitted antibody binding curves, as shown in Figs 3 and 8.

			PFs	
IgG	Preparation	Conformers	EC_50_ (nM)	Max. Signal (A_450nm_)
IVIg	Unfractionated	Mon^1^, Dimer, HMW	286 ± 4.3	1.1 ± 0.1
IVIg	Aβ-isolated	Mon, Dimer, HMW	8.0 ± 0.1	1.4 ± 0.1
		SEC mon^2^	74 ± 1.0	1.7 ± 0.2
		SEC dimers/HMW^3^	1.6 ± 0.1	1.8 ± 0.2
IVIg	Dye-isolated	Mon, Dimer, HMW	22 ± 0.5	1.2 ± 0.1
		SEC mon	104 ± 1.3	1.3 ± 0.0
		SEC HMW	3.3 ± 0.1	1.9 ± 0.3
Avastin	Unfractionated	Mon, Dimer, HMW	1349 ± 113	0.3 ± 0.1
mAb	Aβ-isolated	SEC HMW	11 ± 0.2	0.4 ± 0.1

^1^Mon stands for IgG monomers.

^2,3^SEC-isolated IgG monomers (mon), dimers, and HMW aggregates as shown in Figs 2 & 3.

Cibacron blue-isolated IVIg IgGs bound PFs ~15-fold better than the unfractionated antibody (Fig 3B and Table 2). SEC fractionation of the same samples revealed that dye-isolated, and not unfractionated, IVIg contained HMW aggregates (Fig 3B). SEC fractions of dye-isolated HMW IgG aggregates were ~90-fold stronger at binding to PFs than unfractionated IVIg, with an EC_50_ of 3.3 ± 0.1 nM (Fig 3B and Table 2). Control experiments confirmed that dye-isolated IgG aggregates were not induced by the high salt elution buffer (PBS containing 1.5 M NaCl, pH 7.4) that was used to elute the antibodies off the dye column (Fig 3B). Notably, preparations of dye-isolated IgGs were less enriched in aggregates than Aβ-isolated antibodies, and consequently had ~3-fold lower binding to PFs, with an EC_50_ of 22 ± 0.5 nM (Fig 3B and Table 2). Like Aβ-isolated IVIg monomers, the dye-isolated IVIg monomers were ~8-fold stronger at binding PFs than monomers isolated from unfractionated IVIg, with an EC_50_ of 104 ± 1.3 nM (Fig 3B).

**Fig 4:**
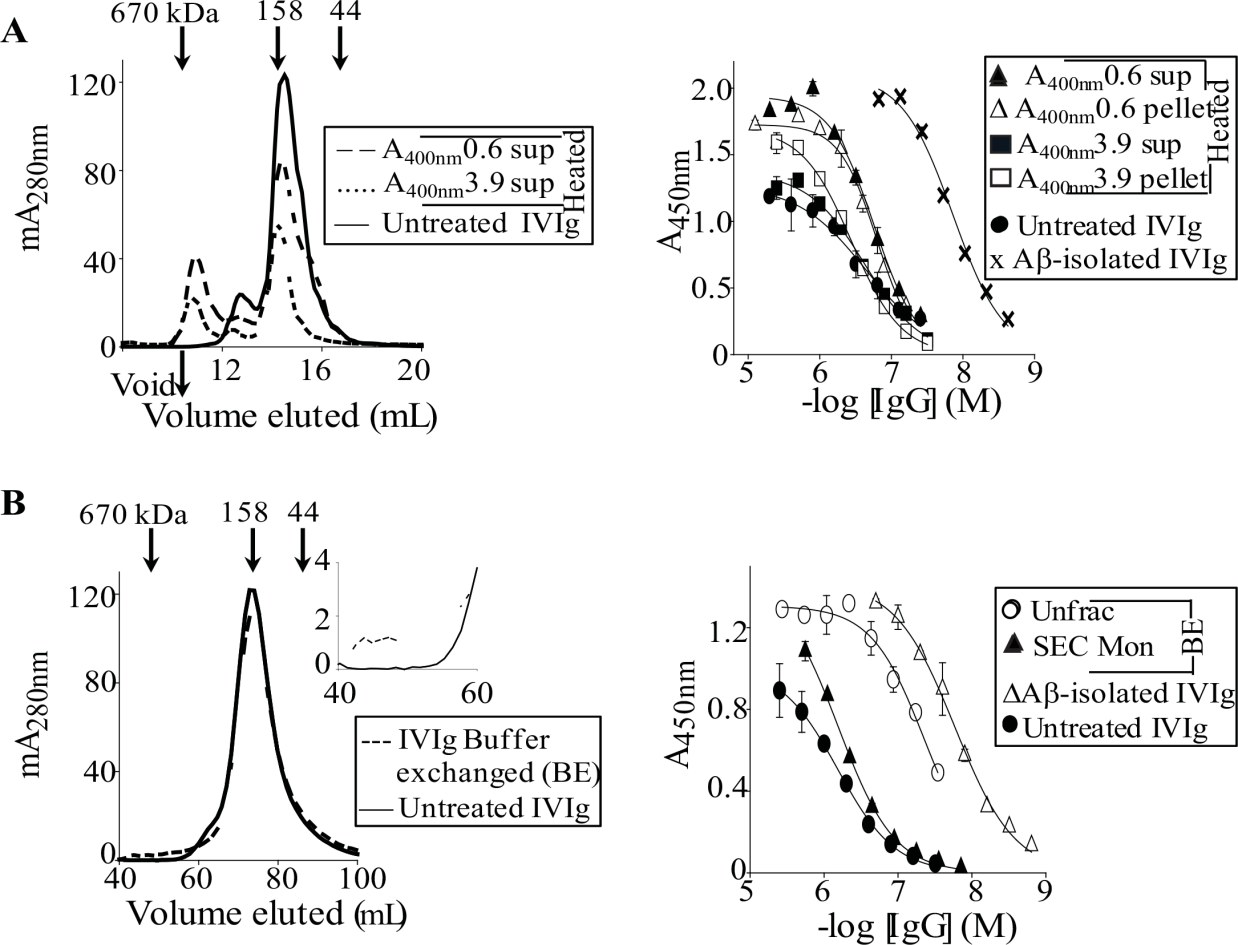
PAb aggregates have diverse avidities for Aβ. (**A**) Left panel: SEC chromatograms for untreated IVIg and for supernatants of ~4 mg/mL of SEC-isolated monomeric IVIg in PBS, pH 7.4, which was heated at 71°C until light scattering at A_400nm_ was 0.6 or 3.9, respectively. Right panel: Antibody binding curves against plate-immobilized PFs for untreated IVIg, Aβ-isolated IVIg IgGs, and for IgG supernatants (sup) and PBS resuspendend pellets (pellet) of heat-treated IVIg. **(B**) Left panel: SEC chromatograms for ~4 mg/mL of untreated IVIg and for IVIg that was buffer exchanged at room temperature from gentle elution buffer (Pierce), pH 6.6, into PBS, pH 7.4. Right panel: Antibody binding curves against plate-immobilized PFs for untreated IVIg, buffered exchanged IVIg that was used unfractionated or as SEC-isolated monomers, and for Aβ-isolated IVIg IgGs.

To further investigate how aggregation of IgGs may give rise to enhanced recognition of PFs, we used heat-treatment to deliberately induce the formation of IgG aggregates. Fig 4A shows that heat-induced IVIg aggregates did not have enhanced avidity for plate-immobilized PFs compared with the native antibody. Nevertheless, maximum PFs binding signals were up to 50% greater for the aggregated than native antibody (Fig 4A). Moreover, we discovered that aggregates formed by buffer exchanging ~2 mg/mL IVIg in high salt buffer (Gentle Elution buffer; Pierce) into PBS had ~20-fold greater avidity for PFs than the untreated antibody, with an EC_50_ value of ~40 nM (Fig 4B). As expected, SEC removal of trace HMW aggregates in preparations of buffer exchanged IVIg abrogated the antibody's enhanced activity (Fig 4B).

**Fig 5:**
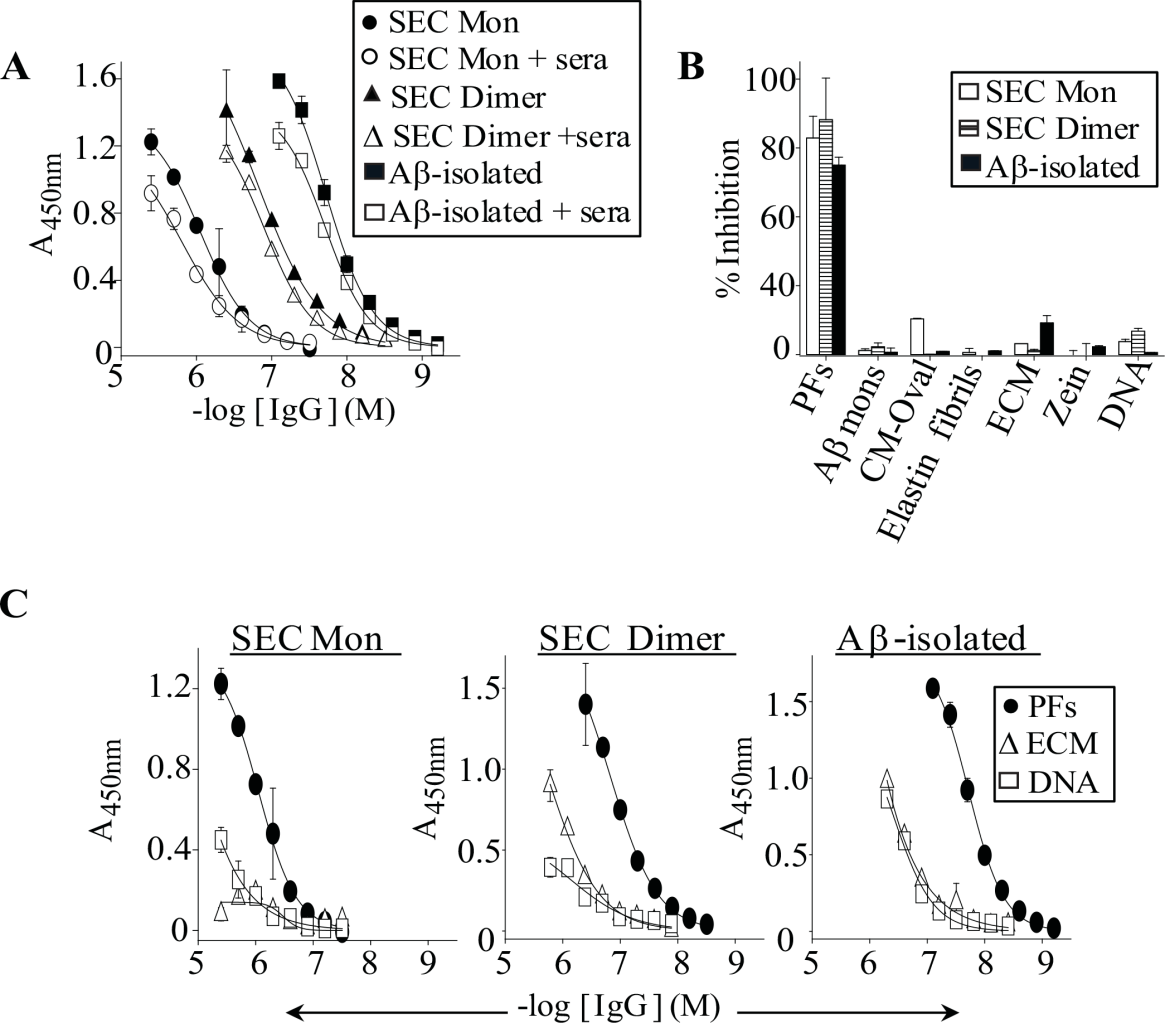
IVIg IgG conformers retain binding to Aβ in the presence of normal human sera and non-amyloid molecules. (**A**) Antibody binding curves against plate-immobilized PFs for SEC-isolated IVIg monomers (SEC Mon), dimers (SEC Dimer), and for Aβ-isolated IVIg IgGs with or without a 1:10 dilution of IgG-depleted normal human sera. Preparations of Aβ-isolated IVIg IgGs contained HMW, dimeric, and monomeric species (see Fig 3A). (**B**) Bar charts for solution-phase PFs, monomeric Aβ and for non-amyloid molecules inhibition of IVIg IgG conformers binding to plate-immobilized PFs. Non-amyloid molecules were chosen based on their: 1) Abundance *in vivo* (extracellular matrix and elastin fibrils), 2) Association with polyreactive autoantibodies (DNA), 3) High hydrophobicity (maize protein zein), and 4) Non-amyloid aggregate state [amorphous aggregated carboxymethylated ovalbumin (CM-Oval)]. Competition studies were carried out using 0.1 mg/mL competitors and concentrations of IgG conformers that were equivalent to their EC_50_ values for PFs: 400 nM IgG Monomers; 50 nM IgG dimers, and 20 nM Aβ-isolated IgGs. Each competition curve was carried out in duplicate, and bars represent the standard error. (**C**) IVIg IgG conformer binding curves against PFs and non-amyloid molecules, murine extracellular matrix gel (ECM) and human DNA.

Having established IVIg's anti-amyloid activity was due to monomeric and aggregate IgG conformers, we determined if the conformers retained activity in the presence of normal human sera or non-amyloid molecules. Fig 5A shows that SEC-isolated IVIg monomers and dimers, and HMW IgG containing Aβ-isolated IVIg IgGs retained binding to plate-immobilized PFs in the presence of IgG-depleted normal human sera. Consistent with the IgG conformer's retention of activity in human sera, the antibodies maintained binding to PFs in the presence of Aβ monomers and non-amyloid molecules (Fig 5B). Non-amyloid molecules that were chosen to investigate the specificities of IgG conformers for PFs was based on their: 1) Abundance *in vivo* (extracellular matrix and elastin fibrils), 2) Association with polyreactive autoantibodies (DNA), 3) High hydrophobicity (maize protein zein), and 4) Non-amyloid aggregate state (amorphous aggregated carboxymethylated ovalbumin). Although IVIg IgG conformers retained binding to PFs in the presence of non-amyloid molecules, SEC-isolated dimers and Aβ-affinity column treated IgG bound stronger to plate-immobilized ECM and DNA than IgG monomers (Fig 5C).

**Fig 6:**
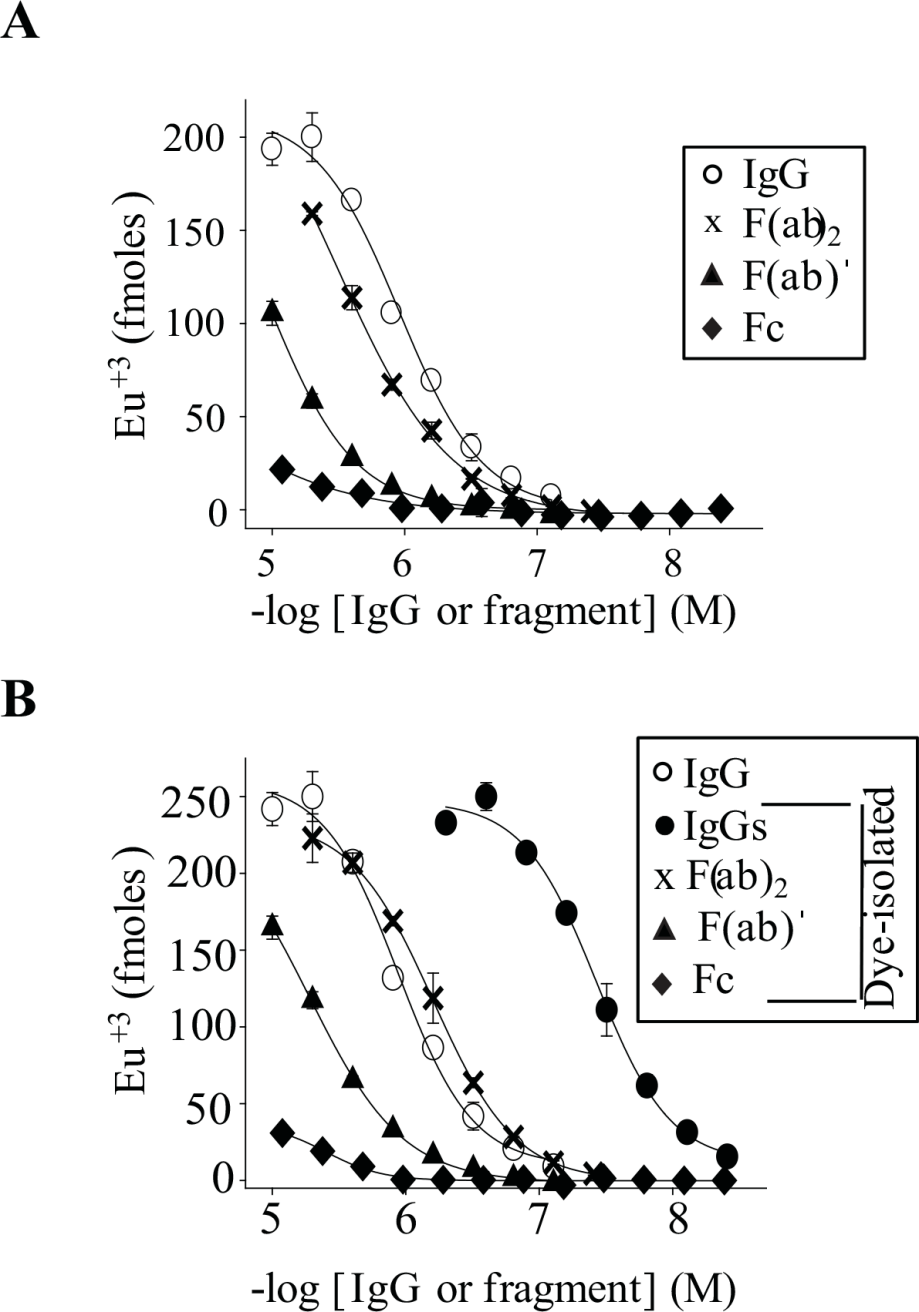
IgG F(ab)ˈs but not Fc mediate IgG binding to PFs. Antibody binding curves are shown against plate-immobilized PFs for intact and fragmented IgGs from preparations of unfractionated IVIg (**A**) and for Cibacron blue-isolated IVIg IgGs (**B**). Antibody binding studies were carried out in triplicate and bars represent the standard error.

To determine which region(s) of IgG molecules bound amyloidogenic conformers, we generated F(abˈ)_2_, F(ab), and Fc fragments of unfractionated and Cibacron blue-isolated IVIg IgGs. Fig 6 shows that F(ab) fragments of unfractionated and dye-isolated IgGs bound similarly to PFs, with EC_50_s of ~10 and ~3 μM, respectively. Likewise, F(abˈ)_2_ fragments of the two antibodies had ~4-fold stronger PFs binding than their F(ab)s, with EC_50_s of ~3 μM and 627 ± **7** nM, respectively (Fig 6). SEC confirmed that ~30-fold stronger PFs binding by dye-isolated IVIg IgGs compared with its F(abˈ)_2_s was due to the presence of aggregates (data not shown). Fc fragments generated from either unfractionated or dye-isolated IVIg IgGs were ineffective at binding to PFs, with EC_50_s >10 μM (Fig 6).

### Anti-amyloid activity is an inherent property of IgGs

**Fig 7:**
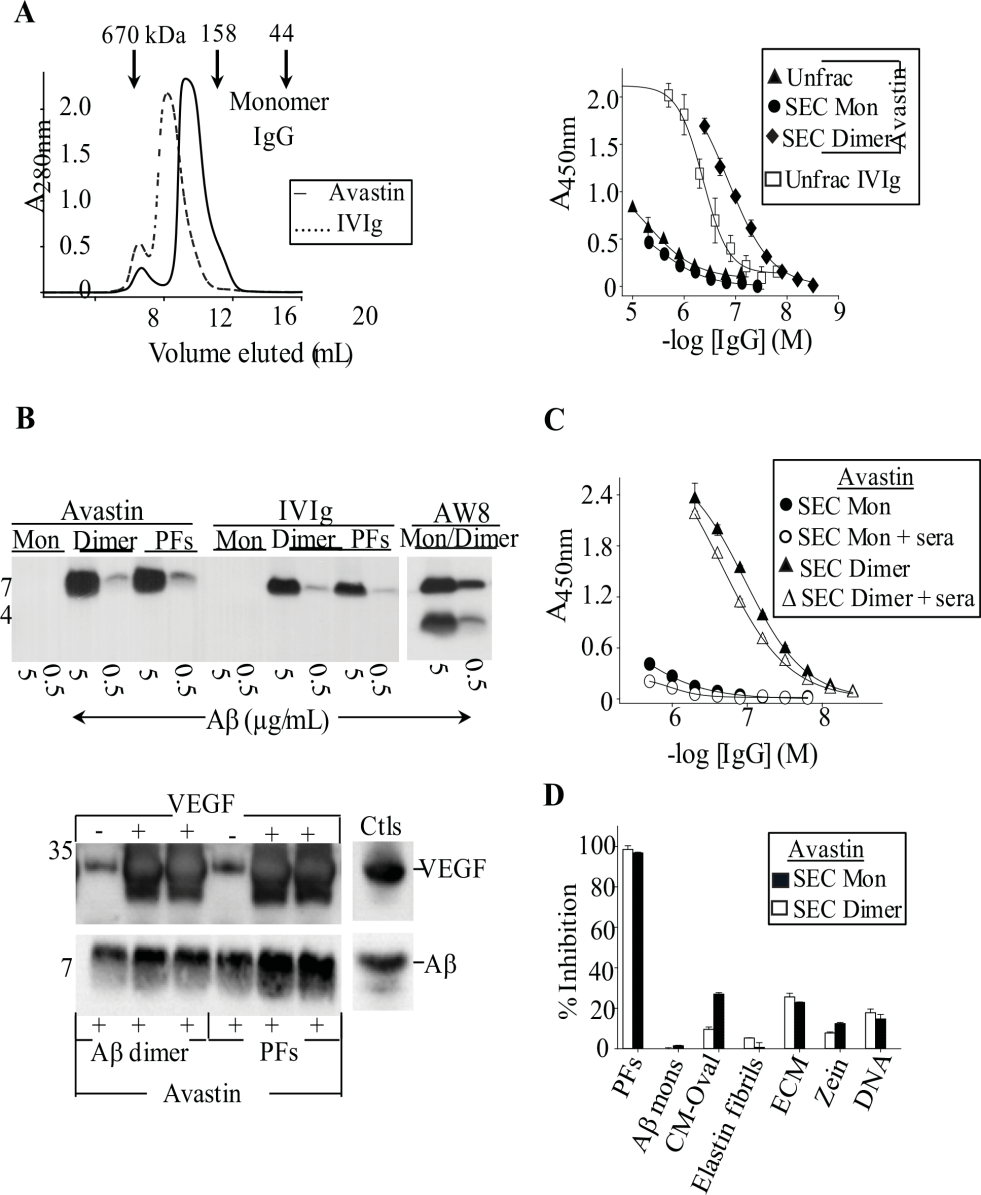
A human mAb generated against a non-amyloid target binds aggregated Aβ. (**A**) Left panel: SEC chromatograms for ~15 mg/mL of mAb Avastin (anti-VEGF) and IVIg. SEC was carried out using a Superdex 200 Increase 10/300 GL column (GE Healthcare) equilibrated in PBS, pH 7.4. Right panel: Antibody binding curves against plate-immobilized PFs for unfractionated (Unfrac) IVIg, and for Avastin used unfractionated or as SEC-isolated monomers and dimers. (**B**) The top Western blots show immunoprecipitation (IP) of synthetic Aβ conformers (monomers (Mon), dimers, and PFs) by 100 μg/mL of Avastin and IVIg, and by 200 μg/mL of a pan-Aβ reactive polyclonal antibody, AW8. The blots were probed for Aβ using an Aβ N-terminal reactive mAb, 6E10 (Signet Laboratories). The lower Western blots show 20 μg/mL mAb Avastin's ability to IP 5 μg/mL of Aβ dimers and PFs in the presence of a 5-molar excess (with respect to Avastin) of a N-terminal 165-amino acid fragment of its immunogen VEGF (VEGF-165). Control IP experiments (Ctls) were carried out using 5 μg/mL mAb 6E10 and a mixture of Aβ dimers and PFs, or with 20 μg/mL Avastin and 1 μg/mL VEGF-165. The blots were probed for Aβ and VEGF-165 using mAb 6E10 and Avastin, respectively. In IPs carried out in the absence of VEGF-165, cross-reactivity of the secondary antibody, goat anti-human IgG (heavy and light, Jackson Immunoresearch Laboratories Inc), with Avastin’s Ig light chain caused a faint band that migrated near VEGF-165. (**C**) Avastin IgG conformers binding curves against plate-immobilized PFs in the presence or absence of a 1:10 dilution of IgG-depleted normal human sera. (**D**) Bar charts for solution-phase PF's, Aβ monomers, and non-amyloid native and aggregated molecule's inhibition of Avastin monomers an dimers binding to plate-immobilized PFs. Competition studies were carried out using 0.1 mg/mL competitors and concentrations of Avastin conformers that were equivalent to their EC_50_ values for PFs: 500 nM IgG Mon, and 200 nM IgG dimer. Each competition curve was carried out in duplicate, and bars represent the standard error.

Given the inherent stickiness of amyloidogenic aggregates [47, 48], we investigated if any human IgG had anti-amyloid activity. Two humanized murine IgG_1_ mAbs generated against non-amyloid targets, Avastin (anti-VEGF, Genentech, Inc.) and Synagis (anti-RSV, MedImmune LLC), recognized Aβ aggregates (Fig 7A, Table 1). The mAbs bound up to 7-fold weaker to plate-immobilized PFs than IVIg, and with up to 8-fold lower maximum signal amplitudes, with EC_50_s of ~1 and ~2 μM, respectively. Like IVIg, the two mAbs recognized aggregated, and not monomeric Aβ (Fig 7B; data not shown), and cross-reacted with soluble TTR aggregates, SAgg, with EC_50_s of ~1 μM (data not shown). Moreover, binding of mAb Avastin to a 5-molar excess of a N-terminal 165 amino acid fragment form of its immunogen (VEGF-165, BioLegend Inc.) did not affect the antibody's ability to immunoprecipitate Aβ dimers and PFs (Fig 7B). Like IVIg, SEC-isolated dimers of Avastin and Synagis were ~15- and ~7-fold stronger than monomers at binding to PFs, and had larger maximum signal amplitudes, with EC_50_s of ~200 nM, respectively (Fig 7 and Table 1). SEC-isolated mAb monomers were ~2-fold weaker at binding to PFs than the unfractionated antibodies, with EC_50_s of ~2 μM (Table 1).

**Fig 8:**
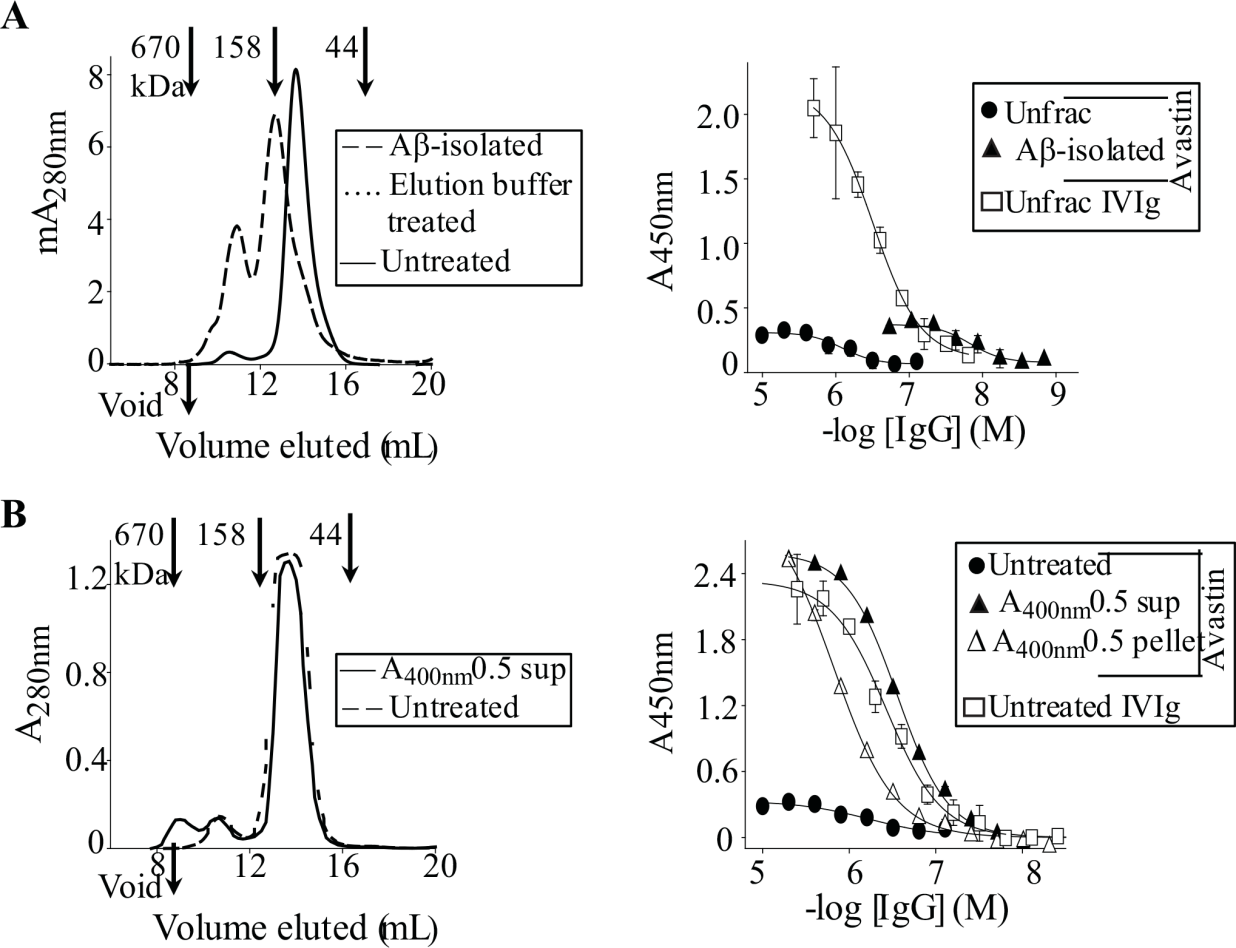
Aβ-isolated but not heat-induced Avastin aggregates have enhanced avidity for PFs. (**A**) Left panel: SEC chromatograms for 0.3 mg/mL of Aβ-isolated Avastin IgGs, untreated Avastin, and for the antibody diluted into elution buffer (0.1 M glycine, pH 2.7) that was used to elute Aβ-bound Avastin IgGs. SEC was carried out using a Superdex 200 increase 10/300 GL column (GE Healthcare) that was equilibrated with PBS, pH 7.4. Right panel: Antibody binding curves against PFs for unfractionated IVIg and Avastin, and for Aβ-isolated Avastin IgGs. (**B**) Left panel: SEC chromatograms for ~5 mg/mL of unfractionated Avastin in PBS, pH 7.4, and for IgG conformers contained in supernatant of 71°C heated Avastin monomers (A_400nm_ 0.5 sup) in PBS, pH 7.4. Right panel: Antibody binding curves against PFs for soluble (A_400nm_ 0.5 sup) and insoluble (A_400nm_ 0.5 pellet) IgG conformers of heat-treated Avastin monomers, and for untreated Avastin and IVIg.

Given the mAb's and IVIg's similar abilities to recognize amyloidogenic substrates, we investigated if Avastin, like IVIg, retained activity in the presence of normal human sera and non-amyloid molecules. Fig 7 shows that SEC-isolated monomers and dimers of Avastin maintained binding to plate-immobilized PFs in the presence of normal human sera or non-amyloid molecules (Fig 7C and 7D). Dose-dependent competition studies with PFs and several non-amyloid competitors confirmed that only PFs were a potent inhibitor of Avastin dimers binding to plate-immobilized PFs, with an IC_50_ of ~3 μg/mL (S2 Fig). Given the mAb's and IVIg's similar specificities for amyloidogenic aggregates, we established if, like a polyclonal antibody, a subpopulation of highly active IgG conformer(s) could be isolated from mAb preparations by Aβ affinity chromatography. We successfully isolated Avastin IgGs off the Aβ column, but the yield (~0.06% of IgGs passed through the column) was ~2-fold lower than for IVIg [27]. Notably, the yield for Aβ-isolated Avastin IgGs was reduced in half (~0.03%) when Avastin column flow throughs were passed through the Aβ column a second time. The latter confirmed that Aβ-isolated Avastin IgGs constituted a discrete antibody subpopulation. The SEC chromatographs in Fig 8A show that Aβ column-isolated Avastin IgGs, like Aβ-isolated IVIg IgGs, contained a substantial amount of aggregates. DLS indicated that the Aβ-isolated Avastin IgG conformers existed as heterogeneous HMW species [*R*_*h*_ = 9.55 nm (26% polydispersity) and 16.9 nm (21% polydispersity), respectively]. However, unlike Aβ-isolated IVIg, Avastin eluants did not contain DLS detectable IgG monomers, although by SEC some IgG molecules eluted near the elution volume for monomeric IgG (Fig 8A).

ELISA studies established that Aβ-isolated Avastin IgGs had ~120- stronger binding to plate-immobilized PFs than the unfractionated antibody, with an EC_50_ of 11 ± 0.2 nM (Fig 8A and Table 2). Notably, Aβ-isolated Avastin and IVIg IgGs had similar avidity for PFs, but the mAb had a ~6-fold lower maximum binding signal (Fig 8B and Table 2). To further investigate PFs binding by Avastin aggregates, we generated heat-induced aggregates. Fig 8B shows that soluble and insoluble conformers of heat-treated Avastin had ~4-fold greater and ~about the same avidity for PFs than the untreated mAb, respectively, with EC_50_s of ~300 nM and ~1 μM. Moreover, the heat-treated mAb had a ~8-fold larger maximum PFs binding signal than was obtained for the untreated IgG (Fig 8B).

Given that both Avastin and Synagis targeted aggregates of Aβ and TTR, we further investigated if amyloidogenic aggregate binding was an inherent property of IgGs using human myeloma mAbs, which were representative of the four Ig isotypes (IgG_1_, IgG_2_, IgG_3_, and IgG_4_). All unfractionated myeloma mAbs recognized PFs, with EC_50_s of ~500 nM, and the IgGs did not target solution-phase Aβ monomers (S1 Fig). Moreover, like the other IgGs tested, SEC-isolated dimers and HMW aggregates of one of the myeloma mAbs had up to ~40- and ~10-fold stronger binding to PFs and SAgg, respectively, than the monomeric antibody (S1 Fig).

## Discussion

### IgG conformer binding to amyloidogenic aggregates

PAbs isolated from pooled normal human plasmas can specifically recognize conformational epitopes on amyloidogenic conformers, and have demonstrated therapeutic potential for amyloid diseases [21, 22, 49]. However, the physiological and clinical significance of pAb's anti-amyloidogenicity is unknown. Presumably, this is in part because pAb preparations contain a complex mixture of IgG conformers (monomers, dimers, HMW aggregates) of naturally occurring and antigen generated antibodies [14, 16, 50]. To address this, we have now established the anti-amyloid activities of IgG conformer's contained in preparations of protein A-purified and commercial (IVIg) pAbs.

Our studies have three main findings. The first observation is that SEC-isolated aggregates of pAbs have greater avidity for aggregates of Aβ and TTR than pAb monomers (HMW species > dimers > monomers). Notably, IgG conformer's retention of binding to PFs in the presence of normal human sera and non-amyloid molecules indicates that this activity may have physiological relevance and clinical significance for amyloid diseases. A second main finding is that great care must be taken when establishing pAb's binding to amyloidogenic aggregates since trace IgG aggregates formed during sample processing can modulate their activity. The last major finding is that IgGs have an inherent ability to recognize amyloidogenic aggregates, and pAb's interactions with amyloidogenic aggregates at least partially involves unconventional (non-CDR) interactions of F(ab)ˈ regions. The latter finding indicates that pAb's anti-amyloidogenicity is not restricted to natural antibodies [51, 52]. Instead, their activity must encompass both naturally occurring IgGs and other antibodies that are generated by antigenic stimulation. Moreover, our demonstration that the F(ab) region of IVIg IgGs was critical for amyloidogenic conformer binding, as well as a recent study implicating their requirement for IVIg's immune modulation of T cell immune diseases [53], suggests that Fab- as well as Fc-dependent mechanisms significantly contribute to IVIg's ability to modulate pathogenic processes.

The stronger binding of aggregated than monomeric IgGs to amyloidogenic conformers indicates that avidity effects (multidentate binding) were crucial. Moreover, similar PFs binding by F(ab) fragments generated from dye-isolated (aggregate-rich) IVIg IgGs and unfractionated IVIg further indicated the central role that avidity effects had on IgG binding to amyloidogenic conformers. Avidity effects alone can strengthen antibody binding to amyloidogenic aggregates, and in doing so, induce preferential antibody binding to aggregated as opposed to monomeric amyloidogenic conformers [54, 55]. However, pAb's preferential binding to amyloidogenic aggregates also likely relies on aggregate-associated conformational epitopes [20, 21, 27]. Moreover, heat aggregated IVIg's and Avastin's lack of enhanced avidity for PFs, indicated that not all IgG aggregates have optimal spacing and/or exposure of amyloid-reactive surface(s) for efficient multi-dentate binding. IgG aggregates with enhanced binding to amyloidogenic conformers seem to be ubiquitously present in IgG products since they were contained in preparations of both IVIg (Gammagard liquid®, Baxter International Inc) and mAbs (Avastin, Synagis, and isotype control myeloma human IgGs). The modulation of IVIg's binding to PFs due to the formation of trace HMW IgG aggregates [56, 57] on buffer exchange of the antibody demonstrated that handling conditions normally sufficient for maintaining IgG activity may not be adequate when establishing the anti-amyloid activity of pAbs. Presumably, this is because pAb's moderate, high nM range, binding to amyloidogenic conformers can be more easily enhanced by trace aggregates than conventional low nM binding of IgGs to antigens. Thus, to ensure that SEC-isolated pAb and mAb conformer's anti-amyloidogenicities were not modulated by contaminating IgG aggregates, we routinely used freshly prepared IgG samples and confirmed their purities with DLS. Notably, it was not necessary to SEC remove trace aggregates in unprocessed IVIg preparations since IgG conformers in these preparations are very stable when stored and handled according to manufacturer's instructions, and they contain only a small amount of IgG aggregates (by our estimates Gammagard liquid®, Baxter International Inc, contained ~5% IgG dimers and <0.1% HMW IgG aggregates).

Given IgG aggregates had enhanced anti-amyloid activity compared with their monomeric forms, it is possible that aggregates formed by other proteins [47, 48] may also have improved binding to amyloidogenic conformers. If so, the latter mechanism does not seem to be a common property of aggregated proteins since, using capture ELSIA, we obtained significantly less PFs binding for aggregated than native forms of two Aβ-reactive proteins: TTR (fibrils and SAgg) [58] and lysozyme (fibrils) [59] (data not shown). Moreover, non-amyloid aggregates formed by elastin fibrils and carboxymethylated ovalbumin did not recognize PFs.

The non-conventional (non-CDRs) component to pAb's binding to amyloidogenic conformers is poorly understood. Nevertheless, this activity seems to be diverse since high salt (PBS containing 0.6 M NaCl versus PBS) strongly inhibited Synagis's but not Avastin's binding to PFs (data not shown). Moreover, the much lower maximum binding signals for mAbs than pAbs against PFs and SAgg indicates that only pAbs had sufficient antibody diversity to saturate all non-conventional IgG binding sites. Alternatively, the larger pAbs binding signals may have been due to contributions from both non-conventional and standard CDR-driven IgG interactions. Presumably, the relatively low enhancement (~40-fold) of Abeta-isolated IVIg IgGs binding to PFs compared with the amount of IgGs that were captured (~0.1 to 0.2% of total IVIg passed through the column [27]) was because a relatively large amount of moderately reactive non-conventional binding IgGs were isolated in addition to high reactive IgGs (primarily aggregates). Our inability to deplete IVIg of moderately Abeta-reactive IgGs after multiple passages of the antibody through an Abeta column indicates that this antibody population constituted a large portion of IVIg and its activity was consistent with IgG's inherent ability to bind to amyloidogenic conformers.

Non-conventional IgG binding to amyloidogenic conformers is analogous to antibody recognition of B-cell super-antigens, nucleotides, catalytic substrates, activated complement components C4b and C3b, and CD4 [60–63]. Although the non-CDR antibody surface(s) that target amyloidogenic conformers is not yet known, V_H_ hinge regions of human IgGs can bind Aβ [64], and V_L_ framework regions (FRs) may target Aβ and non-native TTR in the first step of a split-site mechanism for antibody proteolysis [65–68]. Our findings indicate that the latter mechanism is the most plausible for the inherent anti-amyloid activity of IgGs since PFs were recognized by V_L_ FRs containing, hinge lacking, IVIg F(abˈ)s. Moreover, myeloma mAbs that are representative of the different IgG isotypes have diverse hinge regions but similar binding to PFs.

The moderately stronger PFs binding by dimers and HMW aggregates of pAbs than mAbs may be because pAb conformers were stabilized by end to end F(ab) arm interactions of anti-idiotypic-idiotypic pairs [16] and Fc-Fc interactions [68]. In contrast, mAbs usually form homo- and heterogeneous aggregates that are stabilized by contacts between F(ab)s and/or Fcs [69]. Differential IgG activities may also have been due to differences in antibody sequence, post-translational modification(s), antibody conformational isomerism and/or the IgG's propensities for multi-dentate binding [69–73].

### Physiological significance and clinical utility

Our observations that human IgGs inherently bind to amyloidogenic conformers suggests it is a homeostatic function for clearing and/or neutralizing extracellular amyloid-like misfolded proteins [74, 75]. Evidence for this includes: 1) The retention of pAb and control mAb conformer's anti-amyloidogenicities when dosed into normal human sera and in the presence of non-amyloid molecules (Figs 1, 5 and 7) [27, 76], 2) IgG-Aβ complexes exist in normal human blood and CSF [45, 77, 78], and 3) IgGs isolated from normal human plasma can clear amyloid deposits *in vivo* and improve cognition in certain transgenic mice [21, 49]. Advancing current poor understanding on the molecular basis for IgG's inherent anti-amyloid activity may not only give novel insight in to their physiological functions, but the anti-amyloidogenicity of other Ig-fold containing proteins. Such proteins includes those that are already known to bind amyloidogenic conformers and mediate their cytotoxicity, such as, the receptor for advanced glycation end products (RAGE) [79–83], LilrB2 [84], and FcγRIIb [85].

The clinical utility of the non-conventional anti-amyloid binding of IgGs for amyloid diseases is unknown. Any candidate anti-amyloid therapeutic that is developed would have to be more potent than the anti-amyloid activity of ~10 mg/mL of endogenous IgGs that is present in a patient's blood. One such reagent may be monomeric IgG that is genetically engineered for optimal unconventional binding to amyloidogenic conformers. It remains to be determined if non-conventionally bound amyloidogenic conformers can be cleared by IgGs in the same manner as classically (CDRs) bound forms. For example, in vivo, anti-Aβ IgGs can clear cerebral Aβ through several not mutually exclusive mechanisms, including Fc-mediated clearance, efflux of IgG-Aβ complexes to the periphery, and by a peripheral sink mechanism [86, 87]. Nevertheless, IgGs ability to recognize amyloidogenic conformers in several different ways may be exploited as bifunctional therapeutic molecules, whereby amyloidogenic conformers are bound to the IgG via non-classical interactions, while classical (CDR) binding of the antibody is used to stimulate additional therapeutic pathways, such as the expansion of regulatory T cells [88]

Despite the enhanced binding of IgG aggregates (dimers and HMW species) to amyloidogenic aggregates, these conformers are unsuitable as therapeutic reagents since IgG aggregates are prone to adverse *in vivo* inflammation and off-target binding [89], and consequently are purposely removed during the production of IVIg [90]. Notably, both pAb dimer's and HMW aggregate containing Aβ-isolated IVIg IgG's had greater 'stickiness' than the monomeric antibody against plate-immobilized non-amyloid molecules (Fig 5). Lastly, given a candidate non-conventional anti-amyloid binding therapeutic IgG would recognize common epitopes on amyloidogenic conformers, *in vivo* off-target binding of the monomeric antibody may occur with amyloid deposits that are associated with normal aging [91].

## Supporting Information

S1 Fig Human IgGs have inherent reactivity with amyloidogenic conformers. (**A**) Antibody binding curves against plate-immobilized PFs for IVIg and myeloma mAbs that were representative of the different Ig isotypes, IgG_1_, IgG_2_, IgG_3_, and IgG_4_. (**B**) Representative competition curves for one of the mAb's, IgG_2_, and IVIg, show the antibody's preferential binding to aggregated Aβ, with IC_50_s of ~3 μg/mL. Antibody binding curves against PFs (**C**) and SAgg (**D**) are shown for unfractionated and SEC-isolated conformers of myeloma IgG_2_ mAb. Antibody binding studies were carried out in duplicate and bars represent the standard error.
(EPS)

S2 Fig Solution-phase PFs dose-dependent inhibition of Avastin dimers binding to plate-immobilized PFs. The competition curves show that only PFs was a potent inhibitor of Avastin dimers binding to immobilized PFs. Competition studies were carried out using a concentration of Avastin dimers (200 nM) that was equivalent to its EC_50_ value for PFs.
(EPS)
